# Substrate oxidation and the influence of breakfast in normobaric hypoxia and normoxia

**DOI:** 10.1007/s00421-019-04179-6

**Published:** 2019-07-03

**Authors:** Alex Griffiths, Kevin Deighton, Oliver M. Shannon, Jamie Matu, Roderick King, John P. O’Hara

**Affiliations:** 1 Research Institute for Sport, Physical Activity and Leisure, Leeds Beckett University, Leeds, LS6 3QS UK; 2 Human Nutrition Research Centre, Institute of Cellular Medicine, Newcastle University, Leech Building, Framlington Place, Newcastle Upon Tyne, NE2 4HH UK

**Keywords:** Carbohydrate, Fat, Utilisation, Fasted, Fed, Altitude

## Abstract

**Purpose:**

Previous research has reported inconsistent effects of hypoxia on substrate oxidation, which may be due to differences in methodological design, such as pre-exercise nutritional status and exercise intensity. This study investigated the effect of breakfast consumption on substrate oxidation at varying exercise intensities in normobaric hypoxia compared with normoxia.

**Methods:**

Twelve participants rested and exercised once after breakfast consumption and once after omission in normobaric hypoxia (4300 m: F_i_O_2_ ~ 11.7%) and normoxia. Exercise consisted of walking for 20 min at 40%, 50% and 60% of altitude-specific $\dot{\text{V}}$O_2max_ at 10–15% gradient with a 10 kg backpack. Indirect calorimetry was used to calculate carbohydrate and fat oxidation.

**Results:**

The relative contribution of carbohydrate oxidation to energy expenditure was significantly reduced in hypoxia compared with normoxia during exercise after breakfast omission at 40% (22.4 ± 17.5% vs. 38.5 ± 15.5%, *p* = 0.03) and 60% $\dot{\text{V}}$O_2max_ (35.4 ± 12.4 vs. 50.1 ± 17.6%, *p* = 0.03), with a trend observed at 50% $\dot{\text{V}}$O_2max_ (23.6 ± 17.9% vs. 38.1 ± 17.0%, *p* = 0.07). The relative contribution of carbohydrate oxidation to energy expenditure was not significantly different in hypoxia compared with normoxia during exercise after breakfast consumption at 40% (42.4 ± 15.7% vs. 48.5 ± 13.3%, *p* = 0.99), 50% (43.1 ± 11.7% vs. 47.1 ± 14.0%, *p* = 0.99) and 60% $\dot{\text{V}}$O_2max_ (54.6 ± 17.8% vs. 55.1 ± 15.0%, *p* = 0.99).

**Conclusions:**

Relative carbohydrate oxidation was significantly reduced in hypoxia compared with normoxia during exercise after breakfast omission but not during exercise after breakfast consumption. This response remained consistent with increasing exercise intensities. These findings may explain some of the disparity in the literature.

## Introduction

Disparate metabolic responses have been observed during exercise matched for relative intensities in hypoxia compared with normoxia (Young et al. 1982; Braun et al. 2000; Beidleman et al. 2002; Lundby and Van Hall 2002; Friedmann et al. 2004; Péronnet et al. 2006; Katayama et al. 2010; Morishima et al. 2014; O'Hara et al. 2017; Matu et al. 2017). These contrasting findings within the literature appear to be due to differences in experimental design, specifically pre-exercise nutritional status and exercise intensity (Griffiths et al. 2019). Whilst the effect of pre-exercise breakfast consumption (Edinburgh et al. 2018) and exercise intensity (Van Loon et al. 2001) on metabolism are well documented in normoxic conditions, the metabolic response to these factors is yet to be quantified in hypoxia. In addition, due to the inconsistent use of pre-exercise breakfast consumption in the literature, a direct comparison of the two distinct states during exercise of varying intensities in normoxia and hypoxia may provide clarity on such equivocal findings. Further, whilst the use of studies utilising fasted participants to control for baseline metabolic status is warranted, knowledge of how this differs to fed participants is necessary to generate practical recommendations for relevant populations.

It has been proposed that during exercise matched for relative intensities, the relative contribution of carbohydrate oxidation to energy expenditure is higher in hypoxia compared with normoxia when performed after breakfast consumption, but lower in hypoxia than normoxia when exercise was performed after breakfast omission (Griffiths et al. 2019). A potential explanation of findings observed after breakfast consumption is that greater oxidation and mobilisation of endogenous carbohydrate stores may be stimulated via the combined effect of hypoxia (Katayama et al. 2010) and feeding (Tentolouris et al. 2003) on the sympathetic nervous system. Additionally, a similar effect of hypoxia (Matu et al. 2018) and feeding (Blom et al. 2005) may increase circulating insulin concentration and subsequently inhibit lipolysis and free fatty acid (FFA) mobilisation (Coyle et al. 1997). It also seems plausible that greater fat oxidation may be observed in hypoxia, compared with normoxia after breakfast omission. Increased expression of the transcription factor hypoxia inducible factor 1 alpha (HIF-1α) may upregulate the fatty acid-activated transcription factor peroxisome proliferator-activated receptor alpha (PPARα) as per the metabolic response to hypoxia (Aragones et al. 2008). This response may be further stimulated by the fasted state (König et al. 1999), subsequently inhibiting pyruvate dehydrogenase activity (Huang et al. 2002) and enabling greater mobilisation and oxidation of fat stores (Spriet and Watt 2003).

Exercise intensity was also identified as a significant moderator of substrate oxidation during exercise matched to relative intensities in hypoxia (Griffiths et al. 2019). Specifically, the relative contribution of carbohydrate oxidation to energy expenditure was higher in hypoxia compared with normoxia during exercise performed at higher intensities. This was attributed to the hypoxic effect of both altitude and high intensity exercise, augmenting skeletal muscle hypoxia. The subsequent change in substrate oxidation could, therefore, be explained as per the normoxic response to increased exercise intensity (i.e., reduction in adipose tissue blood flow and lipolysis and/or downregulation of carnitine palmitoyltransferase-1) (Sahlin 1990; Romijn et al. 1993; Van Loon et al. 2001). Alternatively, sympathetic nervous system activity may be potentiated by hypoxia and greater exercise intensities, augmenting glycogenolysis and, therefore, carbohydrate oxidation (Watt et al. 2001).

An investigation into the effects of pre-exercise nutritional status and exercise intensity in hypoxia compared with normoxia may provide clarity on the current literature and facilitate the development of nutritional strategies for high altitude mountaineers and military personnel alike. As such, the purpose of this study was to investigate the effect of breakfast consumption or omission on substrate oxidation during exercise matched for relative intensities in normobaric hypoxia and normoxia. These exercise intensities ranged from 40–60% of altitude specific maximal oxygen uptake ($\dot{\text{V}}$O_2max_). We hypothesised that the relative contribution of carbohydrate oxidation to energy expenditure would be increased during exercise matched to relative intensities in hypoxia compared with normoxia after breakfast consumption, but that this response would not occur after breakfast omission. We also hypothesised that the relative contribution of carbohydrate oxidation would be potentiated with increasing exercise intensities in hypoxia compared with normoxia after both breakfast consumption and omission. Due to the differing metabolic response to hypoxia in females compared with males (Braun et al. 2000), all participants in the present study were male. Inclusion of a female sub-group was beyond the scope of the present study but warrants investigation in future studies.

## Methods

### Participants

Twelve, physically active (structured exercise ≥ 3 times a week), healthy male volunteers (23 ± 3 years, 181.1 ± 6.4 cm, 79.8 ± 13.1 kg) provided written, informed consent to participate in this study. The study received institutional ethical approval (Leeds Beckett research ethics committee, application reference: 46180) and was conducted in accordance with the Declaration of Helsinki. All participants were non-smokers, normotensive, free from food allergies and were not taking any medication. None of the participants had travelled to an altitude of > 1500 m within the previous three months and were all currently residing at an altitude < 500 m.

### Experimental design

**Fig. 1:**
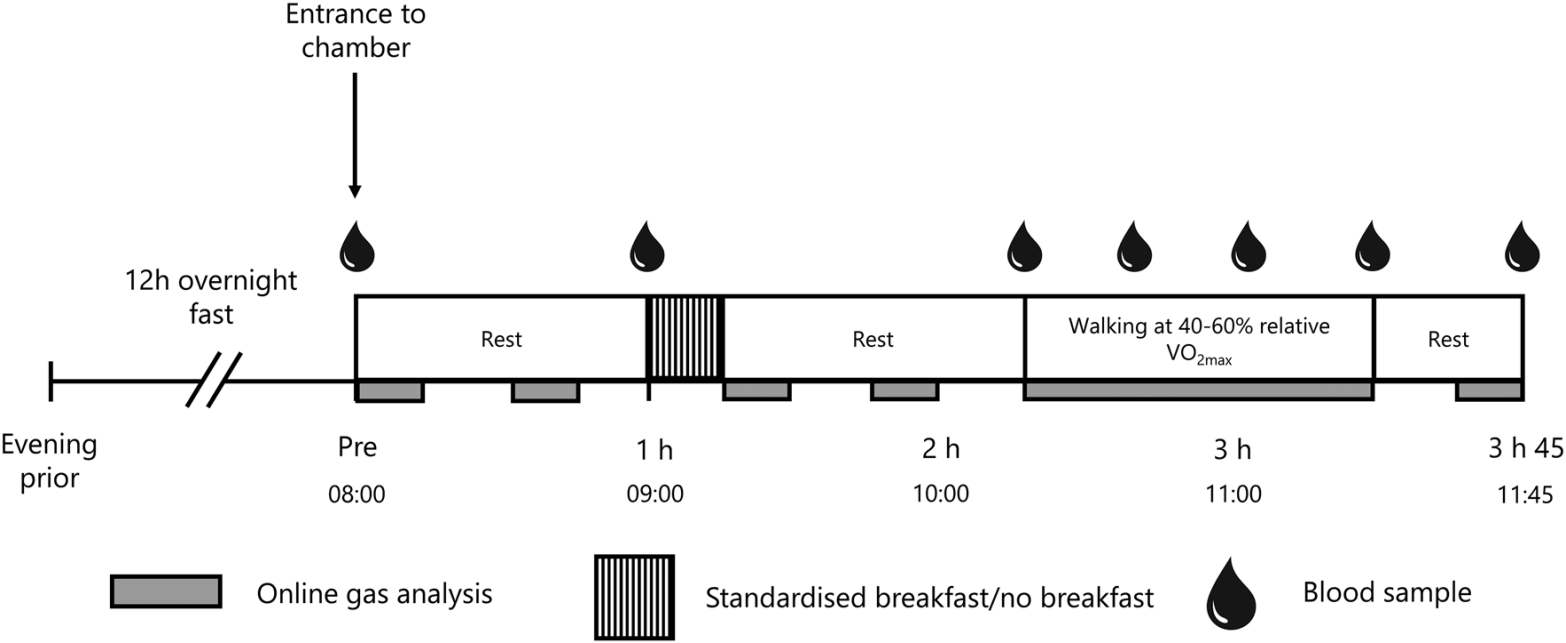
Schematic of full experimental trial. Trials were completed twice in hypoxia (once with breakfast consumption, once with breakfast omission) and twice in normoxia (once with breakfast consumption, once with breakfast omission). Exercise involved 20 min at 40%, 50% and 60% relative $\dot{\text{V}}$O_2max_

Participants were required to make a total of seven visits to the laboratory. The first visit involved pre-exercise screening, anthropometry, verbal familiarisation with testing procedures and a sickle cell trait test. Sickle cell trait was an exclusion criterion due to complications that may occur at altitude, for example, splenic infarction (Goodman et al. 2014). Further exclusion criteria included diabetes and thyroid disorders. The second and third visit required participants to be acutely exposed to normobaric hypoxia (fraction of inspired oxygen (F_i_O_2_): ~ 11.7% when considering water vapour partial pressure (Conkin 2011; Fenn et al. 1946) and daily fluctuations in barometric pressure) equivalent to 4300 m (partial pressure of inspired oxygen (P_i_O_2_): 83 mmHg) in an environmental chamber (TISS, Alton, UK and Sporting Edge, Sheffield on London, UK) or normoxia (absolute altitude ~ 113 m). Ambient temperature was maintained at 20 °C and relative humidity at 50% for all trials. Participants completed sub-maximal and maximal exercise tests to calculate walking speeds matched for relative exercise intensity in each environmental condition for the experimental trials. These two preliminary trials were separated by ≥ 48 h and conducted in a single-blind randomised fashion. On visits 4–7 the participants completed a 3-h 45 min experimental trial, which included a 2-h 15 min rest period, followed by a 1-h incremental walking protocol and a 30-min post-exercise rest period (Fig. 1). Two of the trials were performed in normobaric hypoxia equivalent to 4300 m [one trial with breakfast consumption (HB), and one with breakfast omission (HF)] and two were performed in normoxia [one trial with breakfast consumption (NB), and one with breakfast omission (NF)]. These visits were separated by ≥ 7 days and were randomised independent of the preliminary trials, using a Latin Square design.

### Preliminary testing

Participants completed an exercise test on a motorised treadmill (Woodway PPS S5; Waukesha, WI), which comprised of a sub-maximal and maximal phase. In the normoxic condition, the incremental sub-maximal phase involved four, 3-min stages walking at 3 km/h, 4 km/h, 4.5 km/h and 5.5 km/h. Participants carried a 10 kg backpack to mimic the physiological demands of the experimental trials. The initial two walking speeds in the normoxic condition were performed at a 10% gradient and the second two at a 15% gradient. In normobaric hypoxia, participants walked at 1.5 km/h, 2.5 km/h, 3.5 km/h and 4.5 km/h, at a 10% gradient throughout. Lower speeds and gradients were used in normobaric hypoxia based on the reduced $\dot{\text{V}}$O_2max_ elicited at altitude (Dill et al. 1931), and the need for all participants to achieve 60% $\dot{\text{V}}$O_2max_ within the 12-min trial. The higher gradient utilised in normoxia was employed to ensure participants achieved 60% $\dot{\text{V}}$O_2max_ with a walking gait. Following completion of the sub-maximal phase, participants then rested for approximately 5 min, after which the maximal phase of the test commenced. Within this 5 min, participants were permitted to rest seated, or complete some light stretching. Participants ran without a backpack, at a 1% gradient (Jones and Doust 1996), at a constant speed dependant on fitness and environmental condition, aiming for a perceived exertion of 12. The gradient was increased by 1% every minute until volitional exhaustion. Oxygen uptake ($\dot{\text{V}}$O_2_) and carbon dioxide production ($\dot{\text{V}}$CO_2_) measurements were made throughout both phases of the test using an online gas analysis system (Metalyser, Cortex, Germany), which was calibrated following the manufacturer’s instructions. In this regard, the online gas analyser was calibrated with daily barometric pressure, a 3 L syringe (volume), as well as ambient gases and two known concentrations of gas (15% O_2_ and 5% CO_2_). These gases were subsequently checked before use with an acceptance limit set at ± 0.02% for both O_2_ and CO_2_. All participants were deemed to reach a ‘true’ $\dot{\text{V}}$O_2max_ by fulfilment of > 2 of the following criteria: a plateau in $\dot{\text{V}}$O_2_ in the final exercise stage, respiratory exchange ratio ≥ 1.15, heart rate within 10 b·min^−1^ of age predicted maximum (220-age), rating of perceived exertion (RPE) ≥ 19 and/or blood lactate ≥ 8 mM (Howley et al. 1995). The sub-maximal and maximal data was used to establish walking speeds that would elicit 40%, 50% and 60% $\dot{\text{V}}$O_2max_ relative to both normoxia and hypoxia whilst carrying a 10 kg backpack at a 10% gradient.

### Diet and physical activity before testing

Participants recorded their food intake for the 24 h before the first experimental trial and were instructed to repeat the quantity and timing of this intake for each subsequent visit. During these 24 h, participants were asked not to perform strenuous activity or consume caffeine or alcohol. Participant adherence to these requirements was verbally confirmed before each trial. In addition, on the day before each experimental trial, participants were provided with, and consumed a standardised evening meal at home between 7 and 8 pm that included fusilli pasta, pasta sauce, cheddar cheese, milk and jelly beans (1037 kcal, 57% carbohydrate, 28% fat, 15% protein). This meal was consumed to minimise the possibility of a second meal effect confounding glycaemic control or any other measured variables (Wolever et al. 1988; Stevenson et al. 2005).

### Experimental trials

Participants arrived at the research facilities following a 12 h fast and entered the environmental chamber at 8 am. Verbal confirmation that participants had fasted for the previous 12 h was obtained prior to commencing each trial. Participants then rested for an hour. During rest periods, participants were seated upright and permitted to undertake personal activities such as reading. At 1 h, in both the normobaric hypoxia and normoxia breakfast consumption trials, participants were allowed 15 min to consume a standardised breakfast (535 kcal, 58% carbohydrate, 24% fat, 18% protein). This meal included rolled oats, semi-skimmed milk and orange juice, and was designed to replicate typical breakfast consumption in the UK (Reeves et al. 2013). At 1 h in the normobaric hypoxia and normoxia breakfast omission trials, participants continued resting for 15 min, without the consumption of breakfast. At 1 h 15 min, participants in all trials rested for a further hour. At 2 h 15 min, participants completed a 1-h walking test (20 min at 40%, 50% and 60% $\dot{\text{V}}$O_2max_) at a 10% gradient, carrying a 10 kg backpack, to mimic the demands of high altitude trekking (Mellor et al. 2017). Participants then rested for 30 min after exercise. Water was allowed ad libitum throughout all trials. See Fig. 1 for a schematic representation of the experimental trials.

## Measurements

### Heart rate, capillary oxygen saturation and RPE

Heart rate and capillary oxygen saturation were measured using a fingertip pulse oximeter (Nellcor PM10N, United States) every 15 min during rest. Heart rate, capillary oxygen saturation and RPE were measured every 10-min throughout exercise.

### Expired breath analysis

Expired gas breath samples were collected using an online gas analysis system (Metalyser, Cortex, Germany) for two 10-min resting periods in the first hour (pre-prandial) of exposure (5–15 min and 35–45 min). In the hour following breakfast consumption or omission (post-prandial), a further two 10-min resting periods of expired gas breath samples were collected (1 h 20 min–1 h 30 min and 1 h 50 min–2 h). In addition, these measurements were made continuously throughout the 1-h walking protocol, and for the final 10 min in the 30-min post-exercise period. Participants were fitted with a facemask by researchers 5 min prior to the collection period whilst the participant was seated. At the end of the collection period, participants were asked to remove the facemask to minimise unnecessary opening and closing of the chamber door. This approach (5 min prior to collection and 10 min collection period) has demonstrated reliability previously (correlation coefficient: 0.8) (Spaeth et al. 2015). Coefficient of variation values from the pre-prandial period in the normoxia and normobaric hypoxia trials in the present study (repeated measurements under same conditions) were within 4–9% as recommended by Skinner et al. (1999) (normoxia: $\dot{\text{V}}$O_2_ = 6.2%, $\dot{\text{V}}$CO_2_ = 5.2%; normobaric hypoxia: $\dot{\text{V}}$O_2_ = 4.4%, $\dot{\text{V}}$CO_2_ = 5.2%). Substrate oxidation was calculated using relevant equations for both resting (Frayn 1983) and exercise periods (Jeukendrup and Wallis 2005).

### Blood sampling

Researchers entered the chamber to draw venous blood samples from a 20-gauge cannula (Introcan Safety; B Braun, Sheffield, UK) which was inserted into an antecubital vein upon arrival. Samples were drawn at baseline (before entry to the chamber) for the analysis of plasma glucose, plasma lactate, serum FFA and serum insulin. Subsequent blood samples variables were drawn at 1 h (pre-prandial), 2 h 15 min (post-prandial), 2 h 35 min (40% $\dot{\text{V}}$O_2max_), 2 h 55 min (50% $\dot{\text{V}}$O_2max_), 3 h 15 min (60% $\dot{\text{V}}$O_2max_) and 3 h 45 min (post exercise) (no insulin at this time point due to plate layout). For samples in close proximity (i.e., during exercise), researchers stayed in the chamber to avoid unnecessary opening and closing of chamber doors. Fluoride oxalate tubes used for plasma glucose and lactate were spun at 1500 × *g* for 10 min in a centrifuge (CompactStar CS4, VWR) immediately after being filled with venous blood. The serum separator tubes used for FFA and insulin were spun at the same speed, 30 min after collection to allow for clotting. The supernatant was then transferred into separate Eppendorf tubes to be frozen immediately at − 20 °C before being transferred to − 80 °C until analysis.

### Blood analysis

Commercially available enzyme-linked immunosorbent assay kits were used to determine serum concentrations of insulin (IBL, Hamburg, Germany). To eliminate interassay variation, all samples from each participant were analysed on the same plate. Plasma glucose and lactate, and serum FFA were measured photometrically with reagents from Instrumentation Laboratories (Lexington, MA) and Randox Laboratories (Crumlin, UK). The within batch coefficients of variation were as follows: insulin 5.9%, glucose 1.8%, lactate 2.8% and FFA 3.7%.

### Statistical analysis

Data are expressed as mean ± standard deviation (SD) in text and mean ± standard error (SE) in figures. All data were analysed using IBM SPSS statistics (v24 for Windows; SPSS; Chicago, IL). The trapezoid method was used to calculate area under the curve (AUC) for substrate oxidation and hormone concentrations. The periods of AUC were defined as pre-prandial (0–1 h), post-prandial (1 h 15 min–2 h 15 min), 40% relative $\dot{\text{V}}$O_2max_ (2 h 15 min–2 h 35 min), 50% relative $\dot{\text{V}}$O_2max_ (2 h 35 min–2 h 55 min), 60% relative $\dot{\text{V}}$O_2max_ (2 h 55 min–3 h 15 min) and post exercise (3 h 15 min–3 h 45 min). Normality of distribution was evaluated using histograms and Shapiro–Wilk test and approximated normal distribution. A paired sample *t* test was used to determine differences between $\dot{\text{V}}$O_2max_ in normoxic and hypoxic conditions. One-way repeated measures ANOVA was used to determine differences between trials for energy expenditure, heart rate, capillary oxygen saturation and RPE. Two-way repeated measures ANOVA (time × trial) was used to determine differences between absolute and relative carbohydrate and fat oxidation and hormone concentrations between AUC periods. Where significant main effects of trial were found, further post hoc analysis was performed using Bonferroni correction for multiple comparisons. Effect sizes are presented as Cohen’s *d* and interpreted as ≤ 0.2 trivial, > 0.2 small, > 0.6 moderate, > 1.2 large, > very large and > 4 extremely large (Hopkins 2004).

## Results

### Maximal oxygen uptake and walking speeds

$\dot{\text{V}}$O_2max_ was significantly reduced in hypoxia compared with normoxia (38.3 ± 6.0 mL·kg^−1^·min^−1^ vs. 53.0 ± 8.6 mL·kg^−1^·min^−1^; *p* < 0.001, *d* = 2.00). In hypoxia, this elicited walking speeds of 1.8 ± 0.4 km·h^−1^ (HB: 40.3 ± 4.1%; HF: 38.9 ± 3.1% $\dot{\text{V}}$O_2max_), 2.7 ± 0.5 km·h^−1^ (HB: 47.8 ± 3.3% $\dot{\text{V}}$O_2max_; HF: 48.0 ± 4.4% $\dot{\text{V}}$O_2max_;) and 3.5 km·h^−1^ (HB: 59.6 ± 5.9% $\dot{\text{V}}$O_2max_; HF: 59.1 ± 5.0% $\dot{\text{V}}$O_2max_). In normoxia, this elicited walking speeds of 3.4 ± 0.3 km·h^−1^ (NB: 38.4 ± 3.4% $\dot{\text{V}}$O_2max_; NF: 38.1 ± 3.9% $\dot{\text{V}}$O_2max_), 4.1 ± 0.4 km·h^−1^ (NB: 45.8 ± 3.3% $\dot{\text{V}}$O_2max_; NF: 45.1 ± 2.9% $\dot{\text{V}}$O_2max_) and 4.6 ± 0.5 km·h^−1^ (NB: 61.4 ± 2.5% $\dot{\text{V}}$O_2max_; NF: 61.3 ± 3.3% $\dot{\text{V}}$O_2max_) for each 20-min exercise period. Relative exercise intensity was not significantly different between any trial at 40% (*p* = 0.39), or 60% $\dot{\text{V}}$O_2max_ (*p* = 0.18), however, a trend for an increased relative exercise intensity in hypoxia compared with normoxia after breakfast omission was observed at 50% $\dot{\text{V}}$O_2max_ (*p* = 0.06).

### Experimental trials

#### Energy expenditure

Energy expenditure at rest was significantly greater in hypoxia compared with normoxia in both the breakfast consumption (1252 ± 158 kJ vs. 1108 ± 145 kJ; *p* = 0.02, *d* = 0.95) and breakfast omission trials (1349 ± 250 kJ vs. 1053 ± 140 kJ; *p* = 0.001, *d* = 1.52). Energy expenditure at rest was not significantly different between breakfast consumption and omission in hypoxia (*p* = 0.66, *d* = 0.38) or normoxia (*p* = 0.49, *d* = 0.47).

Energy expenditure during exercise was significantly reduced in hypoxia compared with normoxia after both breakfast consumption (1809 ± 218 kJ vs. 2477 ± 205 kJ* p* < 0.001, *d* = 3.16) and omission (1734 ± 223 kJ vs. 2425 ± 262 kJ, *p* < 0.001, *d* = 2.83). Energy expenditure during exercise was not significantly different between breakfast consumption and omission in hypoxia (*p* = 0.34, *d* = 0.22) and normoxia (*p* = 0.99, *d* = 0.33).

#### Pre-prandial carbohydrate and fat oxidation

In the pre-prandial period, absolute (Table 1) carbohydrate oxidation (*p* ≥ 0.11, *d ≤ *0.86) and its relative contribution to energy expenditure were not significantly different between trials (HB: 43.8 ± 16.8%, HF: 40.8 ± 24.0%, NB: 34.8 ± 16.2%, NF: 38.4 ± 15.5%, *p ≥ *0.63, *d* ≤ 0.54). In the same period, absolute (*p* = 0.99, *d* ≤ 0.42, Table 1) and relative contributions of fat oxidation were not significantly different between trials (HB: 56.2 ± 16.8%, HF: 59.2 ± 24.0%, NB: 65.2 ± 16.2%, NF: 61.6 ± 15.5%, *p* ≥ 0.63, *d* ≤ 0.54).

**Table 1:** Area under the curve (AUC) values for absolute carbohydrate and fat oxidation in all trials

	Exercise											
	Pre-prandial		Post-prandial		40% $\dot{\text{V}}$O_2max_		50% $\dot{\text{V}}$O_2max_		60% $\dot{\text{V}}$O_2max_		Post exercise	
	CHO oxidation (g)	Fat oxidation (g)	CHO oxidation (g)	Fat oxidation (g)	CHO oxidation (g)	Fat oxidation (g)	CHO oxidation (g)	Fat oxidation (g)	CHO oxidation (g)	Fat oxidation (g)	CHO oxidation (g)	Fat oxidation (g)
H breakfast	12.83 ± 5.90	7.20 ± 2.16	16.05 ± 5.23	7.63 ± 1.55	13.54 ± 8.22	7.33 ± 1.54	15.33 ± 5.05	8.53 ± 1.43	21.77 ± 6.42	8.90 ± 1.81	3.40 ± 3.87	3.68 ± 0.94
H fasted	12.70 ± 7.76	7.60 ± 2.72	6.18 ± 5.04	10.70 ± 2.62	6.07 ± 4.92	9.15 ± 2.38	8.63 ± 5.27	10.83 ± 2.70	14.63 ± 5.13	11.70 ± 2.71	0.95 ± 1.45	5.10 ± 3.06
N breakfast	8.38 ± 4.48	6.53 ± 1.29	13.10 ± 5.37	6.55 ± 1.60	18.60 ± 5.98	8.42 ± 1.83	21.67 ± 7.54	10.33 ± 2.28	34.90 ± 12.12	12.00 ± 3.70	4.30 ± 3.01	2.73 ± 0.79
N fasted	9.40 ± 3.64	6.63 ± 1.92	7.10 ± 4.20	7.48 ± 2.15	14.47 ± 6.33	9.98 ± 2.78	16.62 ± 7.28	11.90 ± 3.76	30.72 ± 11.27	13.35 ± 4.99	2.53 ± 1.94	4.53 ± 1.03

*H* hypoxia, *N* normoxia

#### Post-prandial carbohydrate and fat oxidation

In the post-prandial period, absolute carbohydrate oxidation (Table 1) was significantly higher after breakfast consumption, compared with breakfast omission in hypoxia (absolute: *p* < 0.001, *d* = 1.92) and normoxia (*p* = 0.04, *d* = 1.25). In addition, the relative contribution of carbohydrate oxidation was significantly higher after breakfast consumption, compared with breakfast omission in hypoxia (46.2 ± 14.1% vs. 19.6 ± 14.9%, *p* < 0.001; *d* = 1.84), but not normoxia (45.5 ± 15.4% vs. 29.6 ± 16.9%, *p* = 0.14, *d* = 0.99). Absolute carbohydrate oxidation was not significantly different between hypoxia and normoxia after breakfast consumption (*p* = 0.50, *d* = 0.56) or omission (*p* = 0.99, *d* = 0.20). The relative contribution of carbohydrate oxidation was not significantly different between hypoxia and normoxia after breakfast consumption (*p* = 0.99, *d* = 0.05) or omission (*p* = 0.56, *d* = 0.63).

In the same period, absolute fat oxidation was significantly higher after breakfast omission, compared with consumption in hypoxia (*p* < 0.01, *d* = 1.72) but not normoxia (*p* = 0.99, *d* = 0.49). In addition, the relative contribution of fat oxidation was significantly higher after breakfast omission, compared with consumption in hypoxia (80.4 ± 14.9 vs. 53.8 ± 14.1, *p* < 0.001, *d* = 1.84) but not normoxia (70.4 ± 16.9 vs. 54.5 ± 15.4, *p* = 0.14, *d* = 0.99). Absolute fat oxidation was significantly higher in hypoxia compared with normoxia after breakfast omission (*p* < 0.01, *d* = 1.72) but not consumption (*p* = 0.48, *d* = 0.68). The relative contribution of fat oxidation was not significantly different between hypoxia and normoxia after breakfast omission (*p* = 0.56, *d* = 0.63), or consumption (*p* = 0.99, *d* = 0.05).

#### Exercise carbohydrate and fat oxidation

During exercise at all intensities, absolute (Table 1) carbohydrate oxidation was significantly lower in hypoxia compared with normoxia after breakfast omission (40% *p* < 0.01, *d* = 1.49; 50% *p* = 0.01, *d* = 1.27; 60% *p* = 0.001, *d* = 1.96). Absolute carbohydrate oxidation was also significantly lower in hypoxia compared with normoxia after breakfast consumption at 60% $\dot{\text{V}}$O_2max_ (*p* = 0.02, *d* = 1.42), and approached significance at 50% $\dot{\text{V}}$O_2max_ (*p* = 0.06, *d* = 1.00), but not at 40% $\dot{\text{V}}$O_2max_ (*p* = 0.71, *d* = 0.71). The relative contribution of carbohydrate (with the exclusion of 50% $\dot{\text{V}}$O_2max_) was significantly lower in hypoxia, compared with normoxia after breakfast omission (40% *p* = 0.03; *d* = 0.98; 50% *p* = 0.07, *d* = 0.83; 60% *p* = 0.03, *d* = 0.98) but not breakfast consumption at any intensity (all *p* = 0.99, *d* ≤ 0.42). Absolute carbohydrate oxidation was significantly higher after breakfast consumption compared with omission at all intensities in hypoxia (40%: *p* = 0.02, *d* = 1.14; 50%: *p* = 0.001, *d* = 1.30; 60%: *p* < 0.01, *d* = 1.24) but not normoxia (*p* ≥ 0.09, *d* = 0.68). The relative contribution of carbohydrate oxidation was significantly higher after breakfast consumption, compared with omission at all intensities in hypoxia (40%: *p* < 0.01, *d* = 1.20; 50%: *p* < 0.01, *d* = 1.32; 60% *p* = 0.01, *d* = 1.28), but not in normoxia (all *p* ≥ 0.28, *d* ≤ 0.69).

**Fig. 2:**
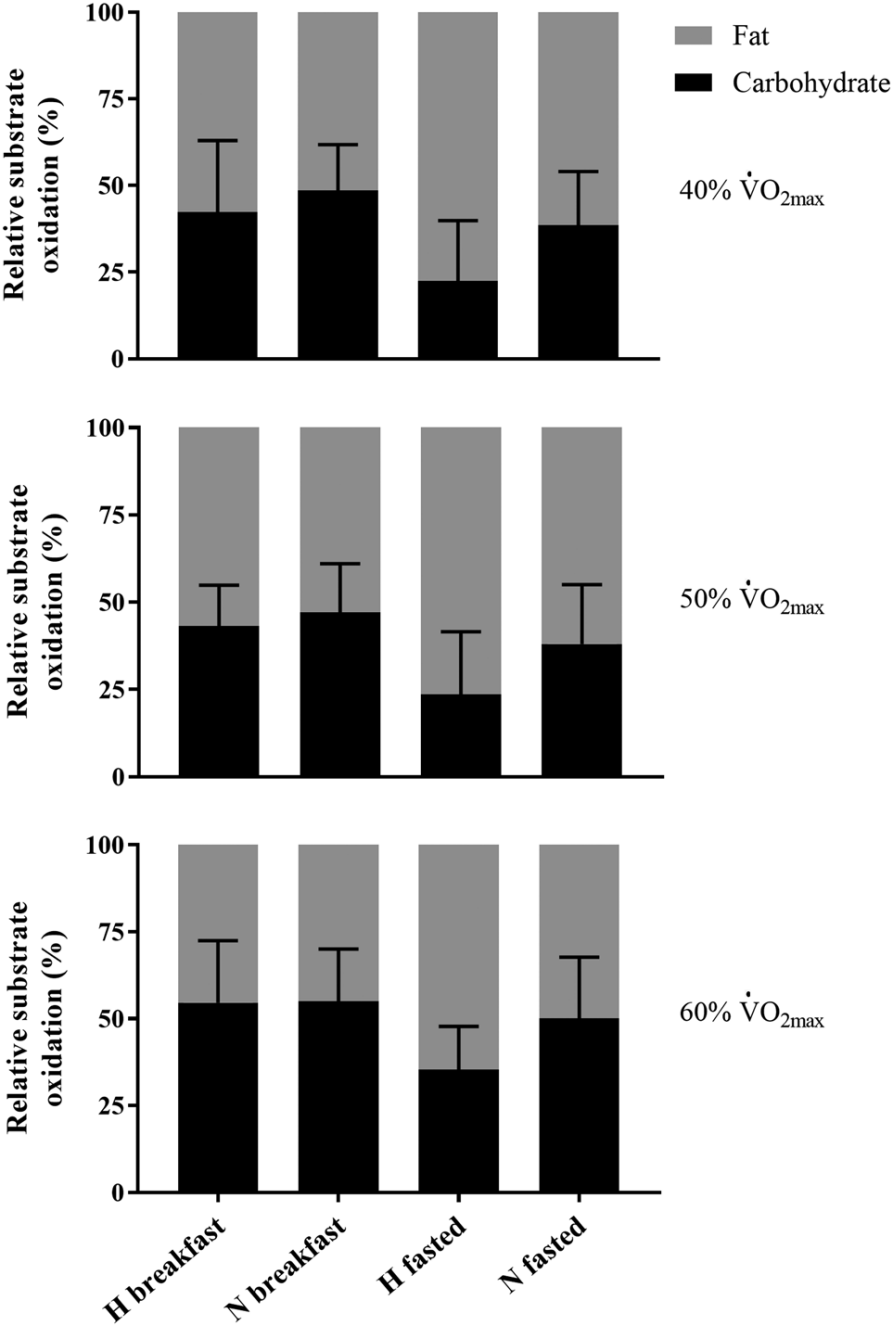
The relative (% energy yield) contribution of carbohydrate and fat oxidation during uphill walking at 40%, 50% and 60% $\dot{\text{V}}$O_2max_ in normoxia and hypoxia after breakfast consumption and omission

During exercise, absolute fat oxidation (Table 1) was not significantly different between hypoxia and normoxia at any exercise intensity after breakfast consumption (*p* ≥ 0.14, *d* ≤ 1.12) or omission (all *p* = 0.99, *d* ≤ 0.43). The relative contribution of fat oxidation (with the exclusion of 50% $\dot{\text{V}}$O_2max_) (Fig. 2) was significantly higher in hypoxia compared with normoxia after breakfast omission (40%: *p* = 0.03, *d* = 0.98; 50%: *p* = 0.07; *d* = 0.83; 60%: *p* = 0.03, *d* = 0.98) but not at any intensity after breakfast consumption (all *p* = 0.99, *d* ≤ 0.42). In addition, absolute fat oxidation was significantly higher at all exercise intensities after breakfast omission compared with consumption in hypoxia (40%: *p* = 0.04, *d* = 0.93; 50%: *p* = 0.02, *d* = 1.12; 60%: *p* = 0.02, *d* = 1.24) but not normoxia (*p* ≥ 0.58, *d* ≤ 0.68). The relative contribution of fat oxidation was significantly higher at all exercise intensities after breakfast omission compared with consumption in hypoxia (40%: *p* < 0.01, *d* = 1.20; 50%: *p* < 0.01, *d* = 1.32; 60%: *p* = 0.01, *d* = 1.28), but not normoxia (*p* ≥ 0.28, *d* ≤ 0.69).

#### Post-exercise carbohydrate and fat oxidation

In the post-exercise period, absolute carbohydrate oxidation was not significantly different between trials (*p* ≥ 0.12, *d* ≤ 0.92). The relative contribution of carbohydrate oxidation was also not significant between trials (HB: 19.9 ± 19.8%, HF: 6.6 ± 10.0%, NB: 30.01 ± 21.2%, NF: 19.7 ± 15.5%, *p* ≥ 0.10, *d* ≤ 1.02). In the same period, absolute fat oxidation was significantly higher after breakfast omission compared with consumption in normoxia (*p* = 0.001, *d* = 1.97), but not hypoxia (*p* = 0.85, *d* = 0.71). Absolute fat oxidation was not significantly different between hypoxia and normoxia after breakfast consumption (*p* = 0.10, *d* = 1.10) or omission (*p* = 0.99, *d* = 0.28). The relative contribution of fat oxidation was not significantly different between any trial (HB: 80.1 ± 19.8%, HF: 93.4 ± 10.0%, NB: 69.9 ± 21.2%, NF: 80.3 ± 15.5%, *p* ≥ 0.10, *d* ≤ 1.02).

#### Blood biochemistry

**Fig. 3:**
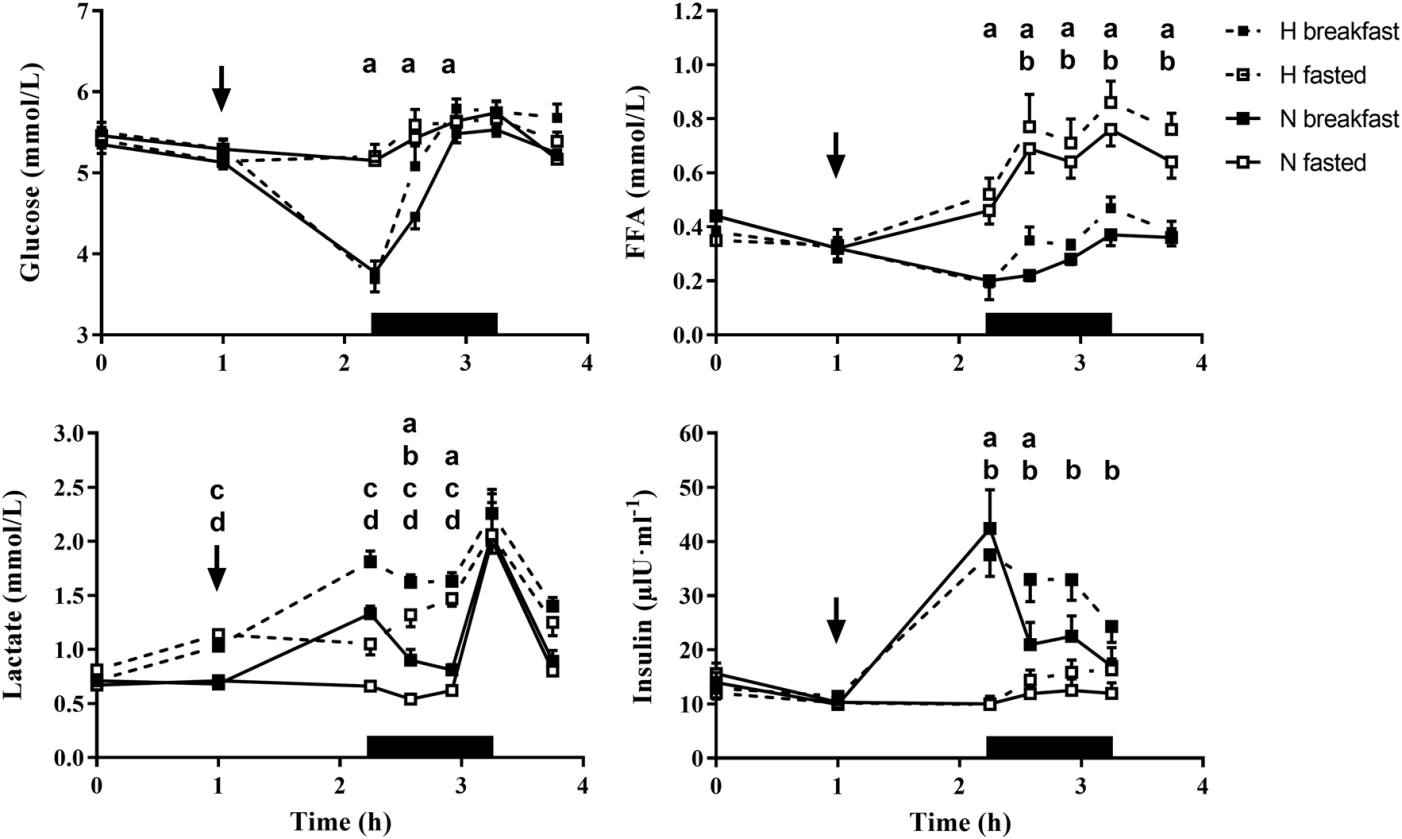
Plasma glucose, plasma lactate, serum FFA and serum insulin concentrations over the full experimental trial. Values are mean ± SE. The thin arrow represents the timing of breakfast in the hypoxic and normoxic breakfast consumption trials. Breakfast was not consumed in the hypoxic and normoxic fasted trials. The black rectangle represents the exercise period. **a** A significant difference between breakfast consumption and omission in normoxia. **b** A significant difference between breakfast consumption and omission in hypoxia. **c** A significant difference between hypoxia and normoxia after breakfast consumption. **d** A significant difference between hypoxia and normoxia after breakfast omission. Significance *p* < 0.05

A significant effect of time (all *p* < 0.01) and trial (all *p* < 0.01) was observed for all analytes. Further, a significant interaction effect of time x trial was also observed for all analytes (all *p* ≤ 0.03). All significant pairwise statistical comparisons are presented in Fig. 3.

#### Heart rate, capillary oxygen saturation and RPE

Capillary oxygen saturation, heart rate, and RPE scores for the duration of the experimental trial are presented in Table 2. There were no significant differences between trials for heart rate (*p* ≥ 0.14, *d* ≤ 0.96) and RPE (*p* ≥ 0.86, *d* ≤ 0.21). Capillary oxygen saturation was significantly lower in hypoxia compared with normoxia in both the breakfast consumption and omission trials (*p* < 0.01, d = 7.83) (*p* < 0.01, *d* = 9.63).

**Table 2:** Mean capillary oxygen saturation (SpO_2_), heart rate and RPE in all trials

	SpO2	Heart rate	RPE
H breakfast	79 ± 3	86 ± 9	12 ± 2
H fasted	80 ± 4	88 ± 21	12 ± 2
N breakfast	79 ± 3	86 ± 9	12 ± 2
N fasted	97 ± 1*	75 ± 13	12 ± 2

*H* hypoxia, *N* normoxia

^*^Denotes significance in comparison with corresponding nutritional status in hypoxia

## Discussion

This study investigated the effect of breakfast consumption and exercise intensity on substrate oxidation in hypoxia compared with normoxia during both rest and exercise. In this regard, the relative carbohydrate contributions to energy expenditure decrease, while relative fat contributions increase, during exercise matched for relative intensities in hypoxia compared with normoxia after breakfast omission, but not consumption. This effect of breakfast consumption in hypoxia compared with normoxia appears to be exclusive to exercise, with no differences in relative substrate oxidation between hypoxia and normoxia after breakfast consumption or omission at rest. Higher exercise intensities did not potentiate carbohydrate oxidation in hypoxia, compared with normoxia after either breakfast consumption or omission.

Absolute carbohydrate oxidation was significantly lower in hypoxia compared with normoxia during exercise at all intensities after breakfast omission. It has long been established that hypoxia induces a lower $\dot{\text{V}}$O_2max_ than normoxia (Dill et al. 1931), which subsequently elicits lower absolute workloads during exercise matched to relative intensities in hypoxia, compared with normoxia (Lundby and Van Hall 2002). As such, the reduction in absolute carbohydrate oxidation in hypoxia, compared with normoxia after breakfast omission is likely due in part to the reduced energy expenditure during exercise in hypoxia. Interestingly, this effect was less pronounced during exercise after breakfast consumption in hypoxia compared with normoxia (significance at 60% $\dot{\text{V}}$O_2max_ only and approaching significance at 50% $\dot{\text{V}}$O_2max_). However, due to the confounding factor associated with utilising absolute substrate oxidation, the use of substrate oxidation data relative to total energy expenditure is warranted to determine an effect of hypoxia beyond a reduced absolute workload.

A novel finding of this study is the lower relative contribution of carbohydrate and higher relative contribution of fat oxidation observed during exercise matched for relative intensities in hypoxia, compared with normoxia after breakfast omission. This finding is in agreement with some existing literature utilising overnight fasted male participants in acute hypoxia (O'Hara et al. 2017) but not others (Katayama et al. 2010; Morishima et al. 2014). O'Hara et al. (2017) observed an increased relative contribution of fat to energy expenditure during exercise matched to relative intensities (74% $\dot{\text{V}}$O_2max_) in hypoxia compared with normoxia. It was proposed that this may in part, be associated with augmented lipolysis and, therefore, FFA oxidation. The increased rates of lipolysis were supported by elevated concentrations of metanephrine and normetanephrine, as well as a subsequent increase in circulating FFA. This effect was observed despite consumption of a carbohydrate drink in both conditions (1.2 g·min^−1^ glucose, 0.6 g·min^−1^ fructose), demonstrating the potency of being fasted during exercise in hypoxia. In the present study, no significant differences in FFA concentrations were observed between hypoxia and normoxia after breakfast consumption or omission. This suggests that the higher relative contribution of fat oxidation observed in hypoxia, compared with normoxia after breakfast omission in the present study may not be associated with increased lipolysis of triglycerides stored in adipose tissue. As such, it seems plausible that an increased oxidation of intramuscular trigylcerides which would not influence circulating FFA concentrations, may contribute to the increased relative contribution of fat oxidation during exercise after breakfast omission in hypoxia, compared with normoxia.

At the mitochondrial level, it has been proposed that the increased relative contribution of fat oxidation to energy expenditure in hypoxia may be a result of increased expression of the transcription factor HIF-1α and the upregulation of PPARα (Aragones et al. 2008). Specifically, PPARα has been demonstrated to deactivate pyruvate dehydrogenase (Huang et al. 2002), inhibiting the conversion of pyruvate to acetyl-coA and, therefore, enabling greater mobilisation and oxidation of fat stores (Spriet and Watt 2003). Subsequently, pyruvate may then be shunted towards lactate production and away from oxidative metabolism. Logically, the reduced relative carbohydrate oxidation observed after breakfast omission in hypoxia, compared with normoxia may be associated with a potentiated PPARα response induced by fasting (König et al. 1999). Therefore, as expected, increased lactate concentrations were observed after breakfast omission in hypoxia, compared with normoxia however, this effect was also evident after breakfast consumption. *Albeit*, lactate concentrations in the fed state may also be inflated by the metabolism of fructose, derived from the consumption of orange juice during breakfast. Specifically, fructose metabolism can occur without the rate limiting step of glycolysis (catalysed by phosphofructokinase) and is, therefore, rapidly phosphorylated leading to increased rates of glycolysis and elevated plasma lactate concentrations (Jentjens et al. 2004; Tappy 2018).

In contrast to findings from our recent meta-analysis (Griffiths et al. 2019), we observed no significant change in the relative contribution of carbohydrate or fat to energy expenditure during exercise matched for relative intensities in hypoxia, compared with normoxia after breakfast consumption. This finding is in accordance with a number of studies investigating substrate oxidation during exercise matched to relative intensities in hypoxia, compared with normoxia (Lundby and Van Hall 2002; Young et al. 1982). However, it is in contrast to those who observed an increased relative contribution of carbohydrate (Péronnet et al. 2006; Friedmann et al. 2004) and fat (Matu et al. 2017; Braun et al. 2000) to energy expenditure in the same conditions. The variance in the literature regarding the use of fed participants is difficult to explain, but may be due to the numerous differing experimental characteristics such as carbohydrate supplementation and the sex of participants. The isolation of each of these characteristics in randomised control trials is required to further understand their influence on substrate oxidation in hypoxia.

Absolute carbohydrate oxidation was significantly higher in the post-prandial period after breakfast consumption compared with omission in both hypoxia and normoxia. Interestingly, the increased absolute carbohydrate oxidation observed after breakfast consumption in hypoxia and normoxia was associated with lower plasma glucose concentrations than breakfast omission. This effect remained significant at 40% and 50% $\dot{\text{V}}$O_2max_ in normoxia and approached significance at 40% $\dot{\text{V}}$O_2max_ in hypoxia. This is likely due to the synergistic effect of augmented insulin concentrations and skeletal muscle contraction during exercise on GLUT-4 trafficking, subsequently inducing an alteration from fat to carbohydrate metabolism (Geiger et al. 2006). Evidence from Edinburgh et al. (2018) suggests that the upregulation of carbohydrate metabolism induced by insulin secretion in the post-prandial state is not solely matched by the subsequent glucose delivery to the muscle, and that for a period before feeding-derived carbohydrate entering the blood stream, muscle glycogen stores are also utilised. Taylor et al. (1993) also observed a reduction in muscle glycogen concentrations 1 h post-meal, before increasing again in the subsequent hours (1–7 h). Whilst we cannot confirm this in the present study, this may have implications for the timing of breakfast before exercise, and also provide a consideration for an additional exogenous carbohydrate source during exercise if the primary goal is to spare muscle glycogen and improve performance.

Alterations in substrate oxidation between hypoxia and normoxia after both breakfast consumption and omission were consistent across varying exercise intensities, suggesting that increasing exercise intensities may not potentiate carbohydrate oxidation in hypoxia, as previously proposed (Griffiths et al. 2019). However, the range of exercise intensities used in the present study were low to moderate intensity (40–60% $\dot{\text{V}}$O_2max_) and, therefore, may not have been sufficient to potentiate sympathetic nervous system activity and glycogenolysis, as plasma epinephrine concentrations have been demonstrated to increase exponentially with exercise intensities increasing up to 85% $\dot{\text{V}}$O_2max_ (Romijn et al. 1993).

The finding that energy expenditure at rest was significantly higher in normobaric hypoxia compared with normoxia is consistent with numerous other studies (Butterfield et al. 1992; Matu et al. 2017). An increase in resting energy expenditure and/or a reduction in energy intake has been associated with a negative energy balance and, therefore, weight loss during chronic hypoxic exposure (Armellini et al. 1997; Sergi et al. 2010). The increase in resting energy expenditure has been attributed to the elevated cardiovascular and ventilatory responses experienced during hypoxic exposure (Butterfield 1999). The reduction in energy intake observed in hypoxia is likely explained by impaired appetite regulation, a result of the hypoxic-induced suppression of the orexigenic hormone, acylated ghrelin (Debevec 2017; Matu et al. 2017). Whilst a reduction in body mass may induce debilitating effects on performance for high altitude mountaineers and military personnel alike, this finding may have implications for weight loss strategies in obese populations (Kayser and Verges 2013). The finding that energy expenditure during exercise was reduced in normobaric hypoxia compared with normoxia substantiates previous research (Matu et al. 2017), and is likely due to the reduced absolute workload in hypoxia. However, this reduced workload in hypoxia has been suggested to induce a similar metabolic load to normoxia, therefore, facilitating physical activity adherence while reducing the risk of musculoskeletal injury in obese individuals (Girard et al. 2017).

Despite the novel findings of this study, some notable limitations must be acknowledged. The use of muscle biopsies to investigate a key theory involving PPARα was beyond the scope of this study and as such, a physiological explanation for the changes in substrate oxidation in the fasted state could not be confirmed. Further, whilst we could calculate whole body substrate oxidation, muscle/liver glycogen oxidation could not be analysed without muscle biopsies or tracer derived methods. In addition, the present study only investigated changes in young men; therefore, caution should be applied when applying the results to other populations. For example, women have been shown to elicit differing metabolic responses to hypoxia, when compared with men (Braun et al. 2000). Finally, the use of a normobaric hypoxic chamber in the present study should be acknowledged when considering the practical applicability of these findings to terrestrial altitude. Future research should investigate the effect of nutritional strategies (i.e., carbohydrate supplementation) in hypoxia after both breakfast consumption and omission to determine their effect on substrate oxidation and performance. In addition, it would be necessary to investigate substrate oxidation during exercise across a broader range of exercise intensities to determine if high intensity exercise induces different substrate oxidation responses than low intensity exercise when performed in hypoxia compared with normoxia.

## Conclusions

In conclusion, we observed a reduced relative contribution of carbohydrate oxidation to energy expenditure during exercise matched for relative intensities in hypoxia, compared with normoxia after breakfast omission but no difference was observed during exercise after breakfast consumption. The effect of hypoxia on substrate oxidation was not altered with increasing exercise intensities when compared with normoxia. These data provide clarity on the current literature and may be useful in the design of nutritional strategies for high altitude mountaineers and military personnel.

## Abbreviations

AUC: Area under the curve
FFA: Free fatty acids
F_i_O_2_: Fraction of inspired oxygen
HIF-1α: Hypoxia inducible factor 1 alpha
P_i_O_2_: Partial pressure of inspired oxygen
PPARα: Peroxisome proliferator-activated receptor alpha
RPE: Rating of perceived exertion
SD: Standard deviation
SE: Standard error
$\dot{\text{V}}$CO_2_: Carbon dioxide production
$$\dot{\text{V}}$$O_2_: Oxygen uptake
$\dot{\text{V}}$O_2max_: Maximal oxygen update
